# ‘What Really Goes on in My Cancer Bubble, They Cannot Understand’: Social Functioning Among Adolescent and Young Adult (AYA) Cancer Patients

**DOI:** 10.3390/curroncol32090501

**Published:** 2025-09-09

**Authors:** Sophia H. E. Sleeman, Milou J. P. Reuvers, Michaela H. van der Veldt, Eveliene Manten-Horst, Olga Husson

**Affiliations:** 1 Dutch AYA ‘Young & Cancer’ Care Network, 1066 CX Amsterdam, The Netherlands; 2 Department of Medical Oncology, Netherlands Cancer Institute—Antoni van Leeuwenhoek, 1066 CX Amsterdam, The Netherlands; m.reuvers@nki.nl; 3 Department of Medical Oncology, Erasmus MC Cancer Institute, Erasmus University Medical Center, 3015 GD Rotterdam, The Netherlands; 4 Department of Psychiatry, Section of Psychosocial Care and Consultative Psychiatry, Erasmus University Medical Center, 3015 GD Rotterdam, The Netherlands; 5 Department of Surgical Oncology, Erasmus MC Cancer Institute, Erasmus University Medical Center, 3015 GD Rotterdam, The Netherlands; 6 Department of Public Health, Erasmus University Medical Center, 3015 GD Rotterdam, The Netherlands

**Keywords:** adolescents and young adults, oncology, social support, social functioning, e-health

## Abstract

Cancer during adolescence and young adulthood (AYA; 18–39 years) can disrupt age-related milestones and impair social functioning. Many AYA patients report unmet social support needs and relationship changes, leading to isolation. This mixed-methods study explores social challenges among AYA patients actively seeking support through a communication tool, the ‘AYA Match app’, supporting communication with loved ones. Upon downloading the app, participants completed questionnaires on social support (MOS-SSS) and social functioning (EORTC CAT) and open-ended questions about social challenges. Eligibility included a first cancer diagnosis at AYA age and fluency in Dutch. The findings show that cancer negatively affected AYA patients’ social functioning. Physical limitations and difficulty relating to peers caused isolation and feelings of loneliness. Some preferred solitude or withheld emotions to protect loved ones. Challenges included forming new relationships, feeling left behind as peers reach milestones, and struggling with a changed life perspective. Participants with children reported less social support. This study highlights the complex social challenges AYA cancer patients face. While support from loved ones is crucial, it may not always be effective. Personalized interventions like peer support, improved family communication, and tailored digital tools are needed to improve social well-being and quality of life in AYAs with cancer.

## Simple Summary

Cancer in young adults (18–39 years) can disrupt important life events and negatively affect social support and relationships. Many young adults with cancer experience loneliness and changes in their social connections. This study examined young adults using the ‘AYA Match’ app, designed to improve communication with loved ones. Findings showed that cancer leads to physical limitations and difficulties in relating to peers, often causing isolation. Some patients chose to be alone or withheld emotions to protect others. They also faced challenges forming new relationships and felt left behind as their peers reached life milestones. Those with children reported lower levels of social support. This study highlights significant social challenges for young adults with cancer. Although support from family and friends is important, it may not always meet their needs. Personalized support—peer groups, improved family communication, and tailored digital tools—may help improve social well-being and quality of life.

## 1. Introduction

Cancer among adolescents and young adults (AYA) is rare: approximately 5% of all cancer patients are between 15 and 40 years old at diagnosis [1]. Cancer has a different impact on the lives of AYA patients compared to pediatric and older adult patients: they find themselves in a life phase that is characterized by maturation in emotional, cognitive, and social domains. A cancer diagnosis might have significant consequences on their ability to have children, complete education, build a professional career, and become (financially) independent. Moreover, AYA cancer patients also report difficulties when it comes to body image, establishing and maintaining relationships, keeping up and connecting with their healthy peers, and sexual and social functioning [2,3,4,5,6,7].

Social functioning is among the most affected health-related quality of life (HRQoL) domains in AYA cancer patients. In comparison to their healthy peers, they have reduced social functioning for at least 24 months after diagnosis [8,9]. Social functioning is defined as “a domain of HRQoL, focusing on limitations and difficulties with participation in social activities and functioning in relationships” [10]. Reduced social functioning is associated with multiple factors among AYA cancer patients: female sex, physical problems, fatigue, (a history of) depression, issues with body image, recurrent thoughts about the disease, the urge for social comparison, experiencing cultural stigma related to cancer, completion of treatment, and a lack of social support. Obtaining sufficient support from their environment and receiving high-quality and age-specific care are factors associated with improved social functioning [9].

House defined social support as “the perception and actuality that one is cared for, has assistance available from other people, and that one is part of a supportive social network” [11]. This comprises multiple aspects, such as psychological support, information, and practical support. Among cancer patients, social support is one of the most helpful factors to cope with the impact of the disease, and receiving adequate social support leads to improved quality of life. A lack of social support can increase psychological issues, such as anxiety or depressive symptoms. Cancer patients often perceive their family and friends as most supportive [12,13], and their support is associated with a positive impact on social well-being and meaningful living [14]. Family universally provides all types of support [13]. For AYA cancer patients, sharing their experiences with cancer peers is also an important source of support, as it reduces loneliness, helps them to feel understood, and allows them to share their worries and fears [15].

However, AYA cancer patients do not always receive adequate social support. They are hesitant to seek support from family and friends because they do not want to burden them, which can result in feelings of isolation [16]. In addition, the relationships with their friends often change because patients are less able to spend time with them, can no longer relate to them, or perceive that their friends act differently around them after diagnosis [13]. AYA cancer patients also reported supportive care needs related to communication with their family, as they often have different coping strategies [17]. The current literature is primarily focused on younger AYAs (<25 years old). This study was conducted to gain insights into the challenges of AYA cancer patients (18–39 years old at diagnosis) who actively sought support through downloading an e-health tool for communication with their loved ones [18]. The purpose of this article is (1) to examine the different challenges these AYA cancer patients experience in daily life concerning social functioning and social support, and (2) identify who is at risk for experiencing these challenges.

## 2. Methods

### 2.1. Participants

A descriptive mixed-methods study was conducted. AYA cancer patients participating in this study were recruited using convenience sampling through the ‘AYA Match app’. This app was developed to support AYA cancer patients and their loved ones in communicating their needs and wishes regarding staying in contact after diagnosis, sending updates to their loved ones, and finding supportive care activities to undertake together [18]. The app was launched during a Dutch AYA congress. It was brought to the attention of patients through healthcare providers, press releases, patient organizations, and hospitals. However, there was no targeted recruitment for the study. When patients voluntarily downloaded the app, they were given the option to participate. This had not been previously communicated by those recommending the app. The application was free to download in the App or Play Store. When opening the app, users were given the choice to participate in the study by completing a questionnaire, or skip this step and continue to use the app. Study eligibility criteria included AYAs diagnosed with any type of cancer for the first time between 18 and 39 years old. In the Netherlands, pediatric cancer care is up to 18 years of age and is centralized, and patients above 18 years old receive age-specific AYA-care. Therefore, we define AYA patients as those diagnosed between 18 and 39 years old. Participants had to be able to read and understand the Dutch language. The study was conducted according to the guidelines of the Declaration of Helsinki and approved by the Institutional Review Board of The Netherlands Cancer Institute (IRBd19-089).

### 2.2. Procedures

All users of the AYA Match App were considered potential participants and were invited via the application’s onboarding screen. The questionnaire was completed prior to using the application. Before starting the questionnaire, an online informed consent form was completed. The administration of the questionnaire took place using the online survey software ‘SurveyMonkey’ (via www.surveymonkey.nl). Data was gathered between 8 March 2018 and 23 April 2021. Completing the questionnaire took approximately 10–15 min. Questionnaire data was separated from a patient’s email address and linked to a study number.

### 2.3. Study Measures

All self-developed items were created in collaboration with various stakeholders, including AYA patients, to ensure the items were relevant and clear.

#### 2.3.1. Demographics and Clinical Data

Demographics and clinical data were derived from a self-developed questionnaire. These questions focused on age, country of residence, gender, (changes in) living situation, (changes in) marital status, parental status, educational level, age at diagnosis, years past diagnosis, type of cancer, treatment phase, and comorbidities.

#### 2.3.2. Social Functioning

Social functioning was assessed using 13 items from the CAT Social Functioning scale from the European Organisation for Research and Treatment of Cancer (EORTC) Quality of Life Group item library [19]. Questions could be answered with responses ranging from “Not at all” to “Very much”, where a higher score indicated better social functioning. Four additional items were added on feeling lonely, others not understanding the impact of the disease, feeling the need to keep worries from loved ones, and finding it difficult to talk about feelings with friends and family. These items were also derived from the item library and were scored similarly. All items were dichotomized as ‘Yes’ (A little to Very Much) or ‘No’ (Not at all).

One open-ended question was developed to determine the impact of cancer on one’s social life. Furthermore, ten items were self-developed to quantitatively measure the impact on patients’ social life. Patients were able to select either ‘yes’ or ‘no’.

#### 2.3.3. Social Support

Social support was measured using the Medical Outcomes Study Social Support Survey (MOS-SSS), including 19 questions. The MOS-SSS was developed by Sherbourne and Steward in 1991 and was used in the Medical Outcomes Study on patients with chronic disease. This questionnaire has high convergent and discriminant validity and is reliable [20]. The MOS items were transferred into four different subscales: emotional support, tangible support, affection, and positive social interactions. Item scores were derived by summing the scores of the items within the subscale. The total score was derived by adding up all 19-item scores. The researchers added one item indicating the number of people in one’s social network.

### 2.4. Data Analysis

Descriptive statistics were used to analyze sociodemographic factors. Independent-sample *t*-tests were used to determine if there were significant differences in social support among patients based on sociodemographic factors (gender, parental status, marital status, educational level, living situation, and social support). The qualitative analysis of the open-ended question was based on the bottom-up thematic analysis from Braun and Clarke [21]. First, all open-question data were reviewed by the two authors (MR, SS), after which all textual information describing any type of social impact was highlighted (open coding). The codes derived from the data were divided into themes, focusing on a specific impact on one’s social life. A subsequent analysis of these codes was conducted to discover overarching themes and subthemes (axial coding). Quotes were inserted to support the data. Quotes were translated from Dutch to English by both researchers to make sure the content did not change in translation. Analyses were performed using SPSS statistical software (version 29, Chicago, IL, USA).

## 3. Results

### 3.1. Demographics

In total, 175 participants completed the questionnaire. First, patients who were not diagnosed within the AYA age range or who had missing information regarding age were excluded (*n* = 25). Next, those who did not complete the questions on social functioning were excluded (*n* = 6). Finally, responses were removed from individuals who completed the questionnaire more than once. It was decided to keep the first response and remove all subsequent ones (*n* = 9), as this meant that the questionnaire was new to participants at the time of completion, and answers could not be based on familiarity with the questions. As a result, the final analysis was conducted on data from 135 participants with a mean age of 29.01 years (SD = 4.19). The characteristics of all participants are reported in Table 1.

### 3.2. Social Functioning

#### 3.2.1. Quantitative Analysis

Several consequences of the cancer diagnosis on the AYAs’ social functioning were reported, including limited participation in social activities due to their disease and not being able to see others as much. Furthermore, many patients liked to be alone instead of being with others (90.4%), but at the same time they indicated that they felt lonely due to a lack of social encounters (63.7–85.2%). Additionally, it appeared that contact with others became more difficult, there was a lack of understanding (80.0%), it was more difficult to share emotions about the illness with those around them (83.0%), and expressing worries was often avoided (87.4%). Elaboration of these consequences is reported in Table 2.

A significant difference was found in the mean social support score (Table 3) between participants with children (M = 47.9, SD = 25.3) and those without children (M = 61.9, SD = 25.1; t (132) = −2.8, *p* = 0.007). No significant differences in social support were found between men and women (*p* = 0.30) or for marital status (*p* = 0.60), living situation (*p* = 0.60), and treatment phase (*p* = 0.80). There was also no significant difference based on educational level mean scores (*p* = 0.80).

More than half of the respondents reported that it was difficult to participate in activities because their disease made social encounters uncomfortable (54.8%) (Table 4). They also felt that they were less easily approached by others (60.7%). Asking others for help and support was also difficult. Nevertheless, some participants reported not receiving adequate help and support from their environment (25.9%).

Thematic analysis of qualitative data among these patients similarly showed the AYAs’ feelings that physical and psychological impairment affected their social life, and resulted in social disconnection.

#### 3.2.2. Qualitative Analysis

A broad variety of challenges in social functioning were reported by the participants. Five overarching themes were identified: physical impairment affecting social life, psychological complaints disrupting social life, social disconnection, limitations in social participation, and no or positive social changes. The themes and subthemes along with codes and quotes are presented in Supplemental Table S1.

Theme 1: Physical impairment affecting social life
Subtheme 1: Being careful due to a compromised immune system

Participants reported that having a compromised immune system due to treatment had a major impact on their social life. Being at high risk of infections prohibited patients from participating in social events and visiting crowded places (1.1.1). In addition, the COVID-19 pandemic significantly contributed to this. The fear of becoming contaminated with the virus during the treatment phase prevented them from leaving their homes (1.1.2). The lack of social gatherings due to the pandemic, on top of their decreased energy levels and becoming fatigued more quickly, resulted in social isolation.

Subtheme 2: Difficulty because of physical discomfort and fatigue

AYA cancer patients experienced major difficulties in managing their social lives as they faced a poor physical condition due to their illness and/or treatment. Dealing with cancer-related fatigue often resulted in skipping social events with friends or having to cut activities short (1.2.1). The lack of energy prevented them from attending family gatherings and seeing friends, making their world smaller and, as a result, increasing the feeling of social isolation and being ‘left out’. Furthermore, side effects from treatment caused physical limitations (e.g., lymphedema and neuropathy), which led to decreased mobility, causing leaving the house to attend social events to be more difficult. As a result, AYA cancer patients felt like their body no longer matched their age and youthful mindset (1.2.2).

Theme 2: Psychological complaints disrupting social life
Subtheme 1: Insecurity and humiliation due to physical change

Participants reported that the disease changed their appearance, such as through hair loss and weight gain (2.1.2). This led to a deterioration in self-esteem and caused leaving the house to become less appealing. Intimacy with their partner also changed, leading to insecurity (2.1.1).

Subtheme 2: Fear, depression, anger, and grief

Negative feelings interfered with the enjoyment of fun activities, for example, panic and fears regarding the future (2.2.1). Furthermore, participants sensed others talking about them behind their backs, which led to staring and being ignored, resulting in insecurity and anger (2.2.2). Negative emotions also occurred due to decreased social functioning. Respondents indicated that they wanted to be normal and remain independent, but that this was impossible, which resulted in frustration.

Subtheme 3: Feeling lonely and being isolated from others

Isolation was often reported and included both feelings of isolation and patients consciously isolating themselves. This was caused by limitations in social activities and having the idea that they were missing out (2.3.1). In addition, their life was different from that of peers, which meant that patients were not able to level with them, and therefore, meeting friends became less appealing. In addition, others did not understand them, could not empathize or did not always sympathize. This resulted in feeling lonely and sometimes caused patients to avoid others or feel different from others. Also, as employment and contact with certain friends was diminished, the patients’ social life was limited (2.3.2).

Subtheme 4: Having little to offer to others (guilt and shame)

Respondents indicated that they were not able to offer their friends, family, and colleagues as much as before. They often felt like they needed to make certain choices in terms of activities they wanted to undertake, as they did not have the energy to participate in all of them. Skipping certain social activities then often led to a sense of guilt and shame (2.4.1). Missing out on things in the future, for example, the life events of their children, also contributed to this guilt and shame (2.4.2).

Theme 3: Social disconnection
Subtheme 1: Not feeling connected to others

Family and friends were often preoccupied with their own lives and, as a result, did not always provide sufficient attention to the patients. Communication was often based on small talk or others talking about things seen as trivial by the patient. Friends passed through certain stages of life that the patient was not going through (yet), such as pregnancy (3.1.1), and sharing this was difficult. The impact of cancer on all kinds of life events, as well as the long-term consequences for the patient, was invisible to their environment. As a result, little attention often was paid to these challenges. There was not always a connection to other patients, given different ages or treatments. The diagnosis often changed the patients’ perspective and outlook on life, while their friends did not go through this change (3.1.2). In addition, others often lived a more carefree life, which created a discrepancy (3.1.3).

Subtheme 2: Decreasing social support over time

Participants spoke of a decrease in social support over the course of their disease and after. At the start, others were very interested, involved, and supportive. Gradually, this often diminished, because others had moved on with their lives more quickly and had processed the shock of the diagnosis (3.2.1). Some patients experienced only minimal contact and did not receive any reaction to updates they shared about their health status (3.2.2). Especially after treatment, the disease was less tangible to others, and many felt that the disease was no longer there or had no impact anymore (3.2.3). In addition, the disease consumed the patient’s life entirely, whereas the illness was only a small part of the life of a friend or family member, resulting in a lack of attention from others (3.2.4).

Subtheme 3: Losing contact and others avoiding you

AYA cancer patients experienced that people close to them (e.g., friends, acquaintances, colleagues) did not know how to deal with the situation, resulting in avoidant behavior (3.3.1). Not being able to count on their loved ones during tough times contributed to feelings of loneliness. Respondents reported a lack of understanding from their surroundings due to difficulties explaining what they were going through. This often resulted in relationships and friendships fading (3.3.2) or even the loss of close friendships (3.3.3).

Subtheme 4: Lack of understanding and empathy and friends making assumptions or staying silent

Conversations were uncomfortable after the diagnosis, as others did not know how to respond when AYAs shared their story, or they received a laconic response: “it will be fine” was oftentimes mentioned. There was often little understanding from friends that the patient does not go through the normative milestones but will follow a different path (3.4.1). On the other hand, loved ones could not always emphasize when a patient tried to live as normally as possible and maintained a positive outlook while going through cancer treatment (3.4.2). In addition, many AYA cancer patients reported that being fatigued after treatment made it more difficult to stay in contact with others. For many friends, this was hard to understand, causing frustration from both sides. These cancer patients found it difficult to share their story and how they were feeling, as they feared what others would say. The impact of the disease (visible and invisible) was oftentimes not clearly evident to their network (3.4.3). Many found that the attention during the diagnosis and initial treatment phase was helpful but diminished after a while. This often meant that they received less support in the phases of processing the impact and dealing with long-term effects. The lack of support and understanding caused AYAs to feel lonely.

Subtheme 5: Disruption of love, romantic relationships, and intimacy

Significant changes in romantic relationships were reported. AYA cancer patients felt burdened and experienced boundaries when it came to dating. Therefore, their love life was disrupted (3.5.1). For AYAs who were in a romantic relationship at the time of their diagnosis, the disease put pressure on the relationship. Couples fought more often (3.5.2) and experienced reduced intimacy. For example, a patient reported that their partner moved out because they could not handle the patient’s cancer-related depression.

Subtheme 6: Setting boundaries, asking for help, and taking others’ advice

Participants indicated difficulties in setting boundaries, asking for help, and accepting others’ advice. As a result, they often did not seek support until problems arose with completing daily tasks, rather than preventing these issues. Also, AYA cancer patients reported that if support was not concrete, it was difficult to accept it, as they did not know what support to prefer (3.6.1). It felt awkward for patients to ask for help with “simple” tasks. Furthermore, pride and a need for independence withheld them from this. For patients, asking for help oftentimes made them feel like they were not able to perform activities by themselves, making them reluctant to request support. For loved ones, this sometimes caused frustration, as they recognized that support was essential to make the patient’s life easier. Well-intended advice was often given but was not perceived as helpful by the patient. Yet, patients were afraid to mention this, as this felt conflicting and they did need help in some ways (3.6.2). Often, patients received unsolicited advice from their surroundings, such as certain alternative therapies.

Subtheme 7: Disrupted/dysfunctional communication

AYA cancer patients often reported difficulties in communicating with the people around them. Friends and acquaintances did not know what to say or how to act around them, which burdened the AYA as they were able to sense this uncertainty. They indicated that it would be more helpful if others would address this issue instead of staying silent and/or distancing themselves (3.7.1). In addition, respondents experienced others talking about them behind their backs instead of directly to them, resulting in not knowing who knew what about their situation (3.7.2). In some cases, existing friendships or contacts turned out to be quite superficial or revolved around more lighthearted affairs (e.g., travel adventures and pregnancies), and therefore, the situation of the patient was neglected or downplayed. This resulted in feelings of loneliness. In addition, AYA cancer patients indicated difficulties in setting boundaries for what they did or did not want to talk about. It was difficult to assess what others wanted to know or when they shared too much. Sometimes, patients experienced conversations which constantly came back to them and their illness, making them feel uncomfortable and ‘abnormal’. In response, they often tried to change the subject (3.7.3). AYA cancer patients sometimes also felt pressure to appear strong and stable, which made them shut others out.

Theme 4: Limitations in social participation
Subtheme 1: Loss of sports and employment

The diagnosis caused many unwanted losses. Many respondents reported being unable to participate in sports during their disease, as they were limited by their physical condition (4.1.1). Several patients reported losing their jobs, which eliminated daily life fulfillment and led to a need to plan other daily activities. Unemployment resulted in a diminished social network, and their world became smaller. Patients also lacked contact with colleagues, or felt that colleagues mostly talked about, instead of to, them because they did not see them as much. There was often little contact after the diagnosis (4.1.2).

Subtheme 2: Lack of social activities due to treatment

AYA cancer patients mentioned that their social lives felt set on hold during their disease, like they were taking a few steps back from their normal social activities (4.2.1). A weakened immune system also led to a decrease in social contact. During the treatment phase, AYA cancer patients were often occupied with hospital visits and taking care of themselves, leading to reduced time to be with friends or family. Aside from that, they felt like they could not participate in all social activities or had to cancel due to unexpected (physical) circumstances (4.2.2).

Subtheme 3: Decreased independency

Participants reported a decrease in their independence in many domains of their lives. Due to the diagnosis, they were not able to perform as many activities as they did before. In addition, having to ask for help was difficult for them, impacting their independence. Patients felt like their freedom was limited because of the disease (4.3.1). Also, loss of mobility made it difficult to go somewhere and to see their loved ones as much. Patients were not able to plan as much for the future, and some had to quit traveling or stay close to home when traveling. Some participants moved back in with their parents, which sometimes caused them to see their friends less often as they were further away. Other patients felt guilty about participating in social activities, since they were on sick leave at work and did not want to be seen by colleagues (4.3.2).

Subtheme 4: Isolation and lack of initiative

AYA cancer patients reported feeling isolated but also dealt with a lack of initiative. It was difficult to initiate an activity, and some preferred to stay at home (4.4.1). This caused them to have a smaller circle and to see friends and family less often. In addition, others’ lives continued (e.g., work, education, family life), inducing a distance between them. Patients experienced feelings of isolation due to their decreased social lives, as they had to stop working, leading to a somewhat empty calendar on weekdays (4.4.2). Participants tended to distance themselves from others (especially groups). Due to the disease, patients were focusing on themselves, trying to recover and rearrange their lives, which caused them not to be involved with others as much as before. Due to low energy, answering text messages was also found to be more difficult, which led to more distance from their loved ones.

Theme 5: No or positive social changes
Subtheme 1: Getting to know your real friends and gaining new friends

Participants reported changes in their friendships. They mentioned that the disease caused them to get to know their real friends and realize who was truly supportive. Others, unexpectedly, appeared to be concerned, resulting in an intensified relationship. The disease also caused them to realize which friendships were truly worth their time and energy and which ones were not (5.1.1). Participating in support groups allowed patients to gain friends, and some existing connections grew stronger. In contrast, there were relationships with friends that deteriorated due to the disease. Since patients no longer participated in daily activities or achieving milestones, their peers found it challenging to stay in contact (5.1.2). Some frineds did not know how to cope with this, which caused friendships to weaken.

Subtheme 2: Increased social support

Some participants experienced increased social support, mainly in the post-diagnosis phase. They reported compassion from others, as well as support and love (5.2.1). Oftentimes this was received from parents, other family members, and friends. Patients received more attention and experienced frequent visits. They realized how much others cared about them and wanted to help. There were many others asking how they were doing (5.2.2).

Subtheme 3: No or positive change

Some AYAs reported no change in their relationships. The disease also caused a positive impact: stronger relationships with their family, valuing life more (5.3.1), being conscious of having to enjoy life, or living in the present moment (5.3.2).

## 4. Discussion

This study provided insight into the challenges AYA cancer patients face related to social support and functioning. Our findings showed that cancer disrupts social interactions with others due to limitations in physical well-being and patients being unable to relate to their peers. This leads to isolation and feelings of loneliness among these patients. Moreover, AYA cancer patients also indicated a preference for spending time alone and wanted to protect their loved ones from fears and worries they experienced, both of which increased feelings of loneliness. They found it difficult to engage in new relationships, such as dating while having a history of illness. In addition, their friends reached age-specific milestones like marriage, extending their family and homeownership, making AYA cancer patients feel left behind. This was emotionally challenging. At the same time, their peers often lacked the life experience necessary to empathize with the patient’s situation. Interestingly, patients with children reported lower levels of social support. Cancer may also alter patients’ outlook on life and expectations of the future, which their healthy peers do not experience. Thus, AYA cancer patients face a “dual crisis” of managing both cancer and normal developmental challenges [22].

A strong social support network is crucial for cancer patients of all ages, as this helps them cope with their illness. While AYA cancer patients often identify family and friends as key sources of support, the way these loved ones respond to the diagnosis is not always perceived as helpful. A study by Pennant et al. highlights unhelpful behaviors, such as treating the patient differently than before or expressing negative emotions. AYAs in romantic relationships or marriages tend to seek support from their partners rather than parents or other family members [23]. They felt like their partners handle their emotions more effectively, whereas parents and other relatives may become overly distressed, offering less constructive support. Providing family members with support systems could help them manage their own emotions, allowing them to offer more stable support when patients share their feelings [23].

AYA cancer patients mentioned specific recommendations to their friends to improve support: seeing each other frequently, not treating them differently, and actively addressing the disease and its impact to gain more insight into how the patient is feeling [23]. Pennant underlined the importance of open communication and the role healthcare providers (HCPs) can have in helping the patient explore and clarify their preferences [23]. As perspectives and assumptions related to receiving support differ between individuals, this should be evaluated and approached accordingly [24]. In addition, these preferences may vary over the course of their disease trajectory, and therefore, HCPs should assess this at multiple times [23]. AYA oncology nurses or social workers could possibly take on this role. In the Netherlands, the majority of hospitals are part of the Dutch AYA ‘Young & Cancer’ Care Network and have an age-specific AYA anamnesis integrated into the electronic patient record (EPR), which also includes themes such as relationships, friendships, emotions, and communication. Dedicated AYA-HCPs have implemented the use of the anamnesis to assess and proactively address the personalized needs of AYA patients throughout the disease trajectory [25,26]. The most crucial aspect of effectively addressing social functioning and social support is a relationship based on trust between the AYA and HCPs [27]. Ideally, an AYA-dedicated HCP should take responsibility in addressing these topics in the first place and determine which HCP is most suitable to further guide the AYA in this regard. For example, this guidance can occur in a primary care setting.

There are interventions that have shown a positive impact on social functioning, including interaction with other young cancer patients, for example, through activities and meetups. Inviting the friends and families of patients to also attend certain events was considered helpful to strengthen their relationship with the patient. When AYAs and their immediate family members struggle to communicate and express their needs to each other, psychotherapy (individualized, dyadic, or group therapy) can be beneficial. Important themes to address, as described by Pennant, include presence, independence, support with daily tasks, positive distraction, maintaining a positive attitude, and communicating with each other [22,23]. Approximately 25% of AYA cancer patients seek information on ways to discuss the disease with their loved ones. Peer support groups might be beneficial for these patients to exchange experiences. Online peer support programs have also been shown to reduce psychological issues, such as distress and anxiety [28]. Furthermore, it decreases social isolation and enhances information-sharing, self-management, coping, and communication skills [8,28]. Since a recent study by Kurisu et al. indicated that AYA patients experiencing loneliness may be at a higher risk for depression compared to older adult patients [29], peer support may be crucial to lower this risk. Our study showed that peer support groups could also result in new friendships. Befriending peers with cancer can be more beneficial than befriending peers without cancer, as AYA cancer patients may want to seek connections with others who have undergone similar experiences [30].

Appropriate peer support might be difficult to establish, as the preferred method of exchange (online or face-to-face) and preferred frequency of meetings differ between patients. Thus, a one-size-fits-all intervention is challenging to implement [15]. For AYA cancer patients in the Netherlands, there is a variety of peer support available. They can join online and face-to-face peer meetings or activities; an overview can be accessed through a nationwide calendar provided by the patient organization “Stichting Jongeren en Kanker”. Loved ones are able to participate in some of these activities as well [31]. This approach enables patients to have autonomy over the role and format of peer support in their lives. Some AYA patients experience obstacles to engage in peer support. They were worried about being in contact with pessimistic patients, befriending someone who might pass away prematurely, or having to listen to emotionally challenging stories. Patients expressed a preference to be able to select peers based on personalized criteria, such as the same stage of disease, age, or family circumstances [32]. This can be offered via a digital application. In the Netherlands, a ‘peer finding tool’ is available on kanker.nl: a centrally initiated Dutch online platform that is structurally funded by the Dutch Cancer Society (KWF), which includes a tool to match cancer patients to appropriate peers [33].

These results must be interpreted in light of the study’s limitations. This study was conducted among individuals who actively sought support for social contact with their surroundings. Therefore, when interpreting the results, possible overestimation of the reported challenges should be considered, as the participants of this study voluntarily downloaded the “AYA Match App”, indicating that they were actively looking for support regarding their social functioning [18,30]. This introduces participation bias, as the participants were likely experiencing social functioning-related challenges to begin with. In addition, since various factors may be interconnected (such as physical symptoms, e.g., fatigue and reduced social activities), future research should provide deeper insight into the interactions between these factors. This could help identify which interventions might best support these patients and pinpoint the exact cause of the issue. Furthermore, this study was conducted among Dutch-speaking cancer patients, as the language used in the application was Dutch. Social impact can differ based on society (e.g., individualistic or collective), healthcare systems, or cultural differences. Additionally, the majority of our study sample consisted of highly educated females, which may affect the results. Moreover, it should be recognized that a large number of participants took part in the study during the COVID-19 pandemic, which may have had an impact on their social functioning. In addition, it should be noted that the self-developed items have not been validated, and their relevance and validity have not been formally assessed. Finally, follow-up research should focus on the impact of parental status on social support, as this study showed lower social support in patients with children. Since we did not have consent to reach out to the participants for more information, more research is needed to look into this.

## 5. Conclusions

This study highlights the complex social challenges that AYA cancer patients experience. A cancer diagnosis at a young age disrupts social relationships and exacerbates feelings of isolation, which are driven by physical impairment, a lack of connection with their peers, and the urge to protect their loved ones from distress. While family and friends are essential sources of support, the effectiveness of their support varies widely, with some interactions even being perceived as unhelpful or distressing. This underscores the importance of HCPs addressing this topic. Furthermore, personalized social support interventions should be facilitated by HCPs to address the dynamic needs of AYA patients. Interventions such as peer support groups, information and tools for friends and family on how to adequately support AYA cancer patients, therapy to improve family communication, and community events with friends and family may help strengthen patients’ social connections. Digital tools that enable tailored peer connections may provide a promising avenue for facilitating appropriate support. These findings contribute to a deeper understanding of the social support complexities faced by AYA cancer patients, though they should be considered in light of the study’s limitations. Future research should focus on adaptable, patient-centered support strategies to improve the social well-being and overall quality of life for young adults navigating cancer.

## Figures and Tables

**Table 1:** Sociodemographic and clinical data of the AYA cancer patients.

	AYAs
	*n* (%)
**Sex**	
Female	112 (83.0)
Male	23 (17.0)
**Marital status**	
Married or living with partner	72 (53.3)
In a relationship, not living together	12 (8.8)
Divorced	1 (0.7)
Single	50 (37.2)
**Children**	
Yes	33 (24.4)
No	102 (75.6)
**Living situation**	
With parents/foster parents	29 (21.5)
Living alone	21 (15.6)
Living with housemates	7 (5.2)
Living with partner/children	71 (52.6)
Other	7 (5.2)
**Educational level**	
Secondary vocational education	10 (7.4)
Higher vocational education	57 (42.2)
University	68 (50.4)
**Age at diagnosis, y**	
18–23	20 (14.8)
24–29	71 (52.5)
30–39	44 (32.7)
**Years past diagnosis**	
Within the first year	52 (38.5)
1–5 years	74 (54.8)
6–10 years	9 (6.7)
Type of cancer *	
Breast	52 (38.5)
Testis	6 (4.4)
Sarcoma	1 (0.7)
Leukemia	9 (6.7)
Hodgkin Lymphoma	15 (11.1)
Non-Hodgkin Lymphoma	8 (5.9)
Brain	7 (5.2)
Melanoma	11 (8.1)
Cervix	9 (6.7)
Colorectal	1 (0.7)
Thyroid	7 (5.2)
Lung	2 (1.5)
Other	11 (8.1)
**Phase of treatment**	
Active	75 (55.6)
Wait-and-see	13 (9.6)
In remission	31 (23.0)
Palliative	6 (4.4)
No treatment plan yet	4 (3.0)
Other	6 (4.4)
Secondary disease interfering with daily functioning *	
None	85 (63.0)
Physical disease	31 (21.4)
Psychological disease	24 (17.8)

* Participants were allowed to select multiple options; percentages do not add up to 100.

**Table 2:** Social functioning among AYA cancer patients.

Did Your Physical Condition, Mental State and/or Medical Treatment Cause You to…	Yes *n* (%)		No*n* (%)
prefer to spend time alone	122	(90.4%)	13 (9.6%)
feel hampered in social activities	121	(89.6%)	14 (10.4%)
tend to hide your worries/fears for your friends or family	118	(87.4%)	17 (12.6%)
feel lonely	115	(85.2%)	20 (14.8%)
have a hard time talking about your emotions with friends or family	112	(83.0%)	23 (17.0%)
be less able to see friends or family	111	(82.2%)	24 (17.8%)
feel like your loved ones don’t understand you	108	(80.0%)	27 (20.0%)
feel no interest in social activities with others	107	(79.3%)	28 (20.7%)
have a hard time contacting others	102	(75.6%)	33 (24.4%)
feel like you didn’t know what to say to friends or family	99	(73.3%)	36 (26.7%)
feel like you have fewer common interests with friends or family	98	(72.6%)	37 (27.4%)
spend less time with friends or family	94	(69.6%)	41 (30.4%)
feel like your relationships are being hampered	91	(67.4%)	44 (32.6%)
feel isolated from others	86	(63.7%)	49 (36.3%)
not look forward to seeing friends or family	83	(61.5%)	52 (38.5%)
get in the way of a family life	74	(54.8%)	61 (45.2%)
argue with friends or family	55	(40.7%)	80 (59.3%)

**Table 3:** MOSSS scores.

	Mean (SD)
Social support	58.6 (25.8)
Emotional support	20.8 (3.1)
Tangible support	11 (1.5)
Affectionate support	8.3 (1.2)
Positive social interactions	8.2 (1.2)
Total social support scale	51 (6.1)

**Table 4:** Impact on social support among AYA cancer patients.

My Diagnosis Caused…	Yes *n* (%)		No *n* (%)
that my loved ones found it difficult to approach me	82	(60.7%)	53 (39.3%)
it to be difficult for me to ask for help	81	(60.0%)	54 (40.0%)
it to be uncomfortable to take part in social activities	74	(54.8%)	61 (45.2%)
others to experience contact with me was uncomfortable	62	(45.9%)	73 (54.1%)
me to have a feeling my loved ones were avoiding specific questions or topics	56	(41.5%)	79 (58.5%)
it to be difficult to get in contact with my loved ones	48	(35.6%)	87 (64.4%)
it to be difficult refusing help from my loved ones	39	(28.9%)	96 (71.1%)
me not to receive the support I need from my loved ones	35	(25.9%)	100 (74.1%)
me to not receive the help I need from my loved ones	21	(15.6%)	114 (84.4%)
None of these statements are applicable	8	(5.9%)	127 (94.1%)

## Data Availability

The data presented in this study are available on request from the corresponding author.

## Supplementary Materials

The following supporting information can be downloaded at: https://www.mdpi.com/article/10.3390/curroncol32090501/s1, Table S1: Quotes supporting the data.
