# Supplementary material for: ‘What Really Goes on in My Cancer Bubble, They Cannot Understand’: Social Functioning Among Adolescent and Young Adult (AYA) Cancer Patients
Source: Curr Oncol. 2025 Sep 9;32(9):501. doi: 10.3390/curroncol32090501 (PMC12468306; doi:10.3390/curroncol32090501)
Supplement: Supplementary file 1 [file curroncol-32-00501-s001.zip › curroncol-3816541-supplementary.pdf]

6. Appendix

Table S1. Quotes supporting the data
.

Theme	*Subtheme*	In-text reference	*Quote*
1<br>Physical impairment affecting social life	*1.1*<br>*Being careful due to a compromised immune system*	1.1.1	*I am very susceptible to infections and diseases, so I cannot expose myself to crowds.*
		1.1.2	*Since the diagnosis, I have been very tired, which limits my activities. Additionally, due to COVID-19, I am more hesitant to leave the house after the diagnosis.*
	1.2<br>*Difficulty because of physical discomfort and fatigue*	1.2.1	*Extreme fatigue hinders my daily life. During activities with friends, I notice that I get tired more quickly and can't keep up for long. Also, I sometimes decline to do something because I am too tired.*
		1.2.2	*I have the body of an old grandma, with a young mind and family.*
2<br>Psychological complaints disrupting social life	*2.1.*<br>*Insecurity and humiliation due to physical change*	2.1.1	*My self-image has deteriorated, and my partner and I are less intimate than before my diagnosis and treatment. I find that very difficult. It makes me very insecure. I also find it challenging to accept that my 67-year-old mother now takes care of most of our household, because I am often not able to, and my partner works full-time.*
		2.1.2	*I feel insecure, especially because there have been changes in my appearance (hair loss, wig, partial loss of eyelashes, weight gain).*
	2.2<br>*Fear, depression, anger and grief*	2.2.1	*The ground fell beneath my feet. I couldn't believe what I heard. I immediately thought of death because cancer is not good, and many people die from it Every day, I find myself overthinking—what if this happens, what if that happens... It’s tough.*
		2.2.2	*The news about my cancer quickly spread in our village. Because of this, I didn't know who knew about it, and I felt very uncomfortable. There are also many people who either stare at me or ignore me, even though you cannot tell by my appearance. I continue to participate in the social activities I used to do before the diagnosis. However, it makes me very insecure, uncomfortable, sad, and angry.*
	2.3<br>*Feeling lonely and being isolated from others*	2.3.1	*The cancer diagnosis has caused me to isolate myself more from my surroundings. My life is put on hold right now while the rest of the world continues. It makes me feel like I’m missing out, and in social situations, I can feel limited because my development and life are paused.*
		2.3.2	*Lost my job. Friends I no longer hear from, or they don't know what to say. Our lives are different now, so you understand each other less. Trust in people is reduced. Lonely.*
	2.4<br>*Having little to offer*	2.4.1	*I feel less connected to my surroundings. Everyday problems that my friends have seem trivial, which makes me feel like I have less to offer to those around me. I feel ashamed of that, too.*

	to others (guilt and shame)	2.4.2	*Due to the treatment, I will physically deteriorate and miss out on things in the lives of my children.*
3<br>Social disconnection	*3.1*<br>*Not feeling connected to others*	3.1.1	*A certain distance has formed, partly because my friends are all getting pregnant, and I notice that both they and I are keeping a distance to avoid any uncomfortable moments.*
		3.1.2	*It has isolated me from my old group of friends, who have mostly moved on while I had to step back. It's difficult to talk to them about what's happened and how it has changed me.*
		3.1.3	*My friends have moved on while I remained stuck, and I also notice that they lead a much more carefree life*
	3.2<br>*Decreasing social support over time*	3.2.1.	*As time passed – the months went by, I was in the rhythm of chemotherapy for half a year and becoming more vulnerable – my friends seemed to have processed the shock for the most part. They appeared to move on, while I was still in a different life. Happy vacation photos on WhatsApp, despite mentioning that I was seriously ill, pregnant friends complaining about nausea, and at the same time, only receiving a short, standard question, "How are you?". In short, sometimes not knowing how to connect with each other anymore.*
		3.2.2	*You notice that in the beginning, you're a sensation. People find you interesting and exciting. After about a month or 1.5 months, people no longer show interest. I also noticed this on the AYA app. I create notifications for updates myself. People read it, but even when I mentioned this week that I was initially supposed to die and the interim results are so good that I'll keep living and even surgeries are not needed, there is no response from the so-called support team. I think the novelty has worn off.*
		3.2.3	*At the beginning, there was a lot of support. But now that treatment is over, there is a belief that I'm perfectly fine, even though that is not the case*
		3.2.4	*I receive a lot of sympathy, but I experience that the attention span is short. What for me is my entire life right now, is for them something that only overlaps a small part of their life, so it doesn't receive the same amount of attention. I can understand this if I put myself in their shoes, but it's also very frustrating because I'm right in the middle of it and constantly dealing with it.*
	3.3<br>*Losing contact and others avoiding you*	3.3.1	*Many friends or acquaintances didn't know what to say or how to act. Some friends were also hesitant to come by.*
		3.3.2	*Many people avoid you, and those you expect to be there for you are not present when it matters. Whether it's due to fear or ignorance, I don't know. I also often felt misunderstood, because it's difficult to explain what you're going through. As a result, some friendships became quieter.*
		3.3.3	*I have lost my best friend.*
	3.4<br>*Lack of understanding and empathy and friends making assumptions or*	3.4.1	*It's like I'm in some kind of bubble: I can see and hear others, and they can see and hear me, but what really goes on in my 'cancer bubble,' they cannot reach, and they cannot understand.*
		3.4.2	*Additionally, people around me often don't understand that I work, and try to live my life as much as possible. They find it 'strange' that I am so optimistic... I frequently hear, 'Does she really have cancer?!' That hurts and cuts right through my soul.*

	staying silent		*Also, the fact that I will never have 'the typical suburban life' makes it difficult for people to maintain friendships and/or contact me.*
		3.4.3	*My social life has been influenced by cancer because I have experienced this life event, and they cannot always fully grasp the impact it has on tangible aspects (appearance, lack of work, finances), as well as intangible ones (uncertainty about the future, the experience of the disease). These are things you cannot fully share with each other. Additionally, support or interest in your situation decreases once you have finished treatment, but that's when the impact of processing and late effects of the treatment hits.*
	3.5 Disruption of love, romantic relationships, and intimacy	3.5.1	*My love life is completely put on hold. The idea of dating again feels like a big step for me.*
		3.5.2	*Frequent arguments with my partner.*
		3.5.3	*Also, my romantic relationship is not the same as it was. We no longer live together because he couldn't handle my depression after the diagnosis.*
	3.6 Setting boundaries, asking for help and taking others' advice	3.6.1	*Some people offer to help, but don't specify their offer. I feel burdened to ask for help in those situations.*
		3.6.2	*Everyday problems that my friends have seem trivial, which makes me feel like I have less to offer to those around me. I feel ashamed of that too. It makes it harder for me to set boundaries. I don't want to hurt people by rejecting well-intentioned help. It feels strange because, in essence, I do want help, but what I really want is someone around who understands me.*
	3.7 Disrupted/dysfunctional communication	3.7.1	*When I occasionally share something, it is generally well-received, but often there are also casual responses like 'oh, that will pass' or 'okay.' I can't do anything with that. It makes me angry inside, but I don't say it out loud. Awkwardness is key in most of the conversations I have. People don't know what to say. I find this very unpleasant, because I do notice it either way. I prefer people telling me they don't know what to say, rather than beating me around the bush.*
		3.7.2	*The news about my cancer quickly spread in our village. Because of this, I didn't know who knew about it, and I felt very uncomfortable. There are also many people who either stare at me or ignore me, even though you cannot tell by my appearance. I continue to participate in the social activities I used to do before the diagnosis. However, it makes me very insecure, uncomfortable, sad, and angry.*
		3.7.3	*Sometimes it's challenging that the conversation always revolves around me and the illness, and I have to actively change the subject myself.*
4 Limitations in social participation	*4.1 Loss of sports and employment*	4.1.1	*I can't do sports anymore.*
		4.1.2	*Some people I don't see any more at all, work is coming to an end, and colleagues only talk about me, instead of to me.*

	4.2<br>*Lack of social activities due to treatment*	4.2.1	*Taking a few steps back in my social life.*
		4.2.2	*I cannot participate or be present everywhere due to chemotherapy and now the surgery. I had to give up a lot in the past year.*
	4.3<br>*Decreased independency*	4.3.1	*I feel less free!*
		4.3.2	*Due to being more fatigued, I do a lot less. It's also challenging to engage in activities related to my work while I am on sick leave, but colleagues still see me (and there's still no visible sign on the outside).*
	4.4<br>*Isolation and lack of initiative*	4.4.1	*Because I easily shut myself off from the outside world, my social life has taken a backseat. I really have to push myself to do something. The threshold is very high, but once I'm around people, I often enjoy myself.*
		4.4.2	*My world is becoming very small. Friends and family are working and going on with their lives, while for me, there's a huge gap in my daily life due to not working. I miss my job, contact with colleagues, and when my partner comes home from work, I don't have much to share because I experience relatively little. Weekends are well-filled with fun activities, but during the week, I have to force myself to create a daily schedule, and I'm still figuring that out.*
5<br>No or positive social changes	*5.1*<br>*Getting to know your real friends and gaining new friends*	5.1.1	*Making choices in my schedule, realizing the value of different relationships and/or who (or what) drains my energy without giving anything in return.*
		5.1.2	*Some relationships have actually strengthened since the diagnosis, while others have faded a bit, because some people are unsure about how to deal with me and the illness. But overall, I have a good social life.*
	5.2<br>*Increased social support*	5.2.1	*Right now, I still am in contact with and receive plenty of help and attention from friends, family, and colleagues. The connection with social life is currently very intense (more intense than before). I can imagine that this may decrease throughout the remainder of my treatment.*
		5.2.2	*Everyone is there for you and wants to do a lot for you. However, they also ask a lot of questions about how, what, where, and when.*
	5.3<br>*No or positive change*	5.3.1	*You learn to appreciate small things and realize what true friendship is.*
		5.3.2	*It was a wake-up call for everyone to live more in the present moment.*
